# Predicting long-term prognosis after percutaneous coronary intervention in patients with acute coronary syndromes: a prospective nested case-control analysis for county-level health services

**DOI:** 10.3389/fcvm.2023.1297527

**Published:** 2023-12-04

**Authors:** Yue Lu, Yaqian Wang, Bo Zhou

**Affiliations:** Department of Clinical Epidemiology and Evidence-Based Medicine, The First Hospital of China Medical University, Shenyang, China

**Keywords:** ACS, PCI, MACEs, predictive nomogram, county (district) medical center

## Abstract

**Purpose:**

We aimed to establish and authenticate a clinical prognostic nomogram for predicting long-term Major Adverse Cardiovascular Events (MACEs) among high-risk patients who have undergone Percutaneous Coronary Intervention (PCI) in county-level health service.

**Patients and methods:**

This prospective study included Acute Coronary Syndrome (ACS) patients treated with PCI at six county-level hospitals between September 2018 and August 2019, selected from both the original training set and external validation set. Least Absolute Shrinkage and Selection Operator (LASSO) regression techniques and logistic regression were used to assess potential risk factors and construct a risk predictive nomogram. Additionally, the potential non-linear relationships between continuous variables were tested using Restricted Cubic Splines (RCS). The performance of the nomogram was evaluated based on the Receiver Operating Characteristic (ROC) curve analysis, Calibration Curve, Decision Curve Analysis (DCA), and Clinical Impact Curve (CIC).

**Results:**

The original training set and external validation set comprised 520 and 1,061 patients, respectively. The final nomogram was developed using nine clinical variables: Age, Killip functional classification III-IV, Hypertension, Hyperhomocysteinemia, Heart failure, Number of stents, Multivessel disease, Low-density Lipoprotein Cholesterol, and Left Ventricular Ejection Fraction. The AUC of the nomogram was 0.79 and 0.75 in the training set and external validation set, respectively. The DCA and CIC validated the clinical value of the constructed prognostic nomogram.

**Conclusion:**

We developed and validated a prognostic nomogram for predicting the probability of 3-year MACEs in ACS patients who underwent PCI at county-level hospitals. The nomogram could provide a precise risk assessment for secondary prevention in ACS patients receiving PCI.

## 1. Introduction

Coronary Heart Disease (CHD) is a major contributor to global health and socioeconomic burdens, especially with the rising age of the world population. As an alarming emergence, CHD is a global public health issue posing a severe threat to human health (1). Despite an increase in the overall CHD burden, age-adjusted CHD mortality is decreasing in developed countries (2, 3). Low- and Middle-Income Countries (LMIC) face a substantial cardiovascular disease burden as urbanization and societal transformation escalate. According to the Report on Cardiovascular Health and Diseases in China data from 2019, rural and urban areas experienced a significant burden of mortality due to cardiovascular disease (CVD), with CVD accounting for 46.74% and 44.26% of all deaths in these respective regions (4). Furthermore, CHD severity appears to follow a reverse socioeconomic gradient in developing nations (5). A prospective analytical study conducted in India discovered notable socioeconomic disparities in access to primary and secondary prevention for Acute Coronary Syndrome (ACS) management (6).

As a life-threatening symptom of coronary heart disease, ACS includes ST-segment Elevation Myocardial Infarction (STEMI), Non-ST-segment Elevation Myocardial Infarction (NSTEMI), and Unstable Angina (UA) (7, 8). A combination of oral medications and interventional procedures are commonly used to manage patients with ACS (9, 10). Percutaneous Coronary Intervention (PCI), which involves balloon dilatation and stenting, is the preferred method of reperfusion therapy for ACS patients (10–12). Despite undergoing PCI, a subset of ACS patients continue to experience Major Adverse Cardiovascular Events (MACEs), including non-fatal myocardial infarction, non-fatal ischemic stroke, death, and bleeding events (13).

Several cardiovascular disease risk and prognosis assessment tools have been established in different populations to guide clinical practice (14–16). Unfortunately, these risk scores are limited by their short follow-up durations and may not accurately reflect the prognosis of ACS patients, particularly in economically disadvantaged regions where long-term risk stratification for post-PCI ACS patients is unclear. Furthermore, the Human Development Index (HDI) is strongly associated with the CHD prevalence in developing countries, and the current allocation of healthcare resources to county-level hospitals in LMIC is relatively low. Therefore, risk assessment for long-term postoperative surveillance should be simple and suitable for remote follow-up to ensure the continued enhancement of quality healthcare services (17, 18).

Consequently, our study aimed to validate a prognostic model capable of predicting the likelihood of experiencing MACEs in ACS patients who underwent PCI at a county-level hospitals. We employed a multicenter external validation strategy to serve as a reference for the swift screening of high-risk individuals and early clinical intervention. Moreover, our study aimed to foster the advancement of quality healthcare in regions with limited healthcare resources.

## 2. Material and methods

### 2.1. Study population and design

From September 2018 to August 2019, a prospective nested case-control study was undertaken on a primary cohort of ACS patients enrolled for PCI at six county-level hospitals within Liaoning Province, China. Across all study centers, 1,795 individuals were diagnosed with ACS and treated with PCI based on their clinical conditions. A total of 1,741 patients were followed up from recruitment to August 2022, and 3.0% of cases (*n* = 54) were removed due to incomplete clinical information and exclusion criteria. A total of 1,581 patients were finally analyzed after three years of follow-up. The study enrolment and results are shown in Supplementary Figure S2. The original training set used for the prognostic evaluation was constructed using three centers (*n* = 520) that were similar to one another in terms of spatial and socioeconomic features (Supplementary Figure S1). Additionally, the expected sample size was calculated based on 20 events per variable (EPV), and the model was prospectively evaluated using three other cohorts (*n* = 1,061).The case group consisted of 143 ACS patients who experienced a major adverse cardiovascular event during the follow-up period from September 2018 to August 2019. On the other hand, the control group included patients who did not experience an adverse cardiovascular event until the end of the follow-up period.

The following were the inclusion criteria: (1) Patients with ACS who met the Chinese Medical Association's guidelines for diagnosis and treatment of acute coronary syndromes (supplementary methods contain definitions for each subset of ACS); (2) Three years following PCI, patients with complete revascularization. The exclusion criteria were as follows: (1) Patients with hematologic disease, multiple organ failure, or a cancer diagnosis; (2) Patients with significant comorbidity, trauma, or surgery; (3) Patients with incomplete hospitalisation registration information that cannot be followed up; (4) Patients who died within 30 days.

The study was approved by the institutional review board of the First Hospital of China Medical University [approval number: (2019) 189]. Written informed consent was obtained from all surviving participants or the next of kin who provided information about the deceased participants.

The study adheres to the Transparent Reporting of a Multivariable Prediction Model for Individual Prognosis or Diagnosis (TRIPOD) reporting guidelines (19).

### 2.2. Clinical endpoint and definitions

The primary endpoint of this study was MACEs, defined as a composite of stroke, heart failure, target lesion revascularization, recurrent myocardial infarction, and all-cause death.

### 2.3. Data collection and follow-up

The data collected in our study were obtained from scans at various hospitals, and not all participating hospitals were connected to a common electronic health record (EHR) system. Our research team prioritized data privacy and security. We ensured adherence to ethical and legal standards during our data extraction procedures while also implementing robust quality control measures to uphold accuracy and consistency.

Baseline characteristics (patient demographic data, medical history, preoperative clinical characteristics, coronary angiography features, laboratory indicators, echocardiography indices, and medication use during hospitalization) of the training set and external validation set are shown in Table 1.

**Table 1:** Demographic and clinical characteristics of ACS patients in the training and external validation set.

Variable	Training set (*N *= 520)		External validation set (*N* = 1,061)		*P*-value
	MACEs (*N* = 143)	Controls (*N* = 377)	MACEs (*N* = 230)	Controls (*N* = 831)	
Age, years, M (Q1, Q3)	67.0 (61.0, 74.0)	62.0 (55.0, 66.0)	68.0 (63.0, 73.0)	62.0 (55.0, 68.0)	0.828
Sex, (%)					0.045
Male	91 (63.6)	259 (68.7)	127 (55.2)	531 (63.9)	
Female	52 (36.4)	118 (31.3)	103 (44.8)	300 (36.1)	
Admission status, (%)					<0.001
Emergency department	25 (17.5)	88 (23.3)	76 (33.0)	287 (34.5)	
Outpatient department	118 (82.5)	289 (76.7)	154 (67.0)	544 (65.5)	
Days, M (Q1, Q3)	9.0 (7.0, 12.0)	8.0 (7.0, 10.0)	8.0 (5.0, 13.0)	7.0 (5.0, 11.0)	<0.001
ACS, (%)					<0.001
STEMI	74 (51.7)	155 (41.1)	77 (33.5)	277 (33.3)	
NSTEMI	24 (16.8)	95 (25.2)	77 (33.5)	251 (30.2)	
UA	45 (31.5)	127 (33.7)	76 (33.0)	303 (36.5)	
Killip III-IV, (%)	12 (8.4)	6 (1.6)	14 (6.1)	9 (1.1)	0.176
Hypertension, (%)	103 (72.0)	234 (62.1)	162 (70.4)	503 (60.5)	0.441
Diabetes, (%)	49 (34.3)	108 (28.6)	60 (26.1)	238 (28.6)	0.418
Dyslipidemia, (%)	29 (20.3)	48 (12.7)	68 (29.6)	177 (21.3)	<0.001
HHcy, (%)	10 (7.0)	6 (1.6)	39 (17.0)	92 (11.1)	<0.001
Arrhythmia, (%)	40 (28.0)	76 (20.2)	51 (22.2)	123 (14.8)	0.005
AF, (%)	8 (5.6)	7 (1.9)	11 (4.8)	23 (2.8)	0.849
PAD, (%)	7 (4.9)	16 (4.2)	13 (5.7)	24 (2.9)	0.438
HF, (%)	35 (24.5)	44 (11.7)	20 (8.7)	27 (3.2)	<0.001
CI, (%)	14 (9.8)	9 (2.4)	11 (4.8)	29 (3.5)	0.626
PHD, (%)	3 (2.1)	0 (0.0)	5 (2.2)	1 (0.1)	1.000
Number of PCI, (%)					<0.001
≤1	141 (98.6)	368 (97.6)	212 (92.2)	772 (92.9)	
>1	2 (1.4)	9 (2.4)	18 (7.8)	59 (7.1)	
Smoking, (%)	53 (37.1)	144 (38.2)	68 (29.6)	259 (31.2)	0.006
Alcohol consumption, (%)	22 (15.4)	84 (22.3)	35 (15.2)	148 (17.8)	0.148
HR, bpm, M (Q1, Q3)	74.0 (65.0, 82.0)	72.0 (63.0, 81.0)	71.0 (64.0, 80.0)	72.0 (64.0, 82.0)	0.691
SBP, mm Hg, M (Q1, Q3)	140.0 (130.0, 156.0)	140.0 (126.0, 158.0)	140.0 (127.0, 160.0)	140.0 (125.0, 154.0)	0.610
DBP, mm Hg, M (Q1, Q3)	80.0 (74.5, 90.0)	82.0 (80.0, 98.0)	85.50 (80.0, 97.0)	82.00 (79.0, 92.0)	0.978
Previous stroke, (%)	31 (21.7)	66 (17.5)	53 (23.0)	112 (13.5)	0.137
Previous PCI, (%)	12 (8.4)	39 (10.3)	28 (12.2)	63 (7.6)	0.477
Previous MI, (%)	28 (19.6)	65 (17.2)	54 (23.5)	124 (14.9)	0.633
TA, (%)	3 (2.1)	15 (4.0)	1 (0.4)	8 (1.0)	<0.001
Thrombolysis, (%)	14 (9.8)	33 (8.8)	23 (10.0)	100 (12.0)	0.146
MDP, M (Q1, Q3)	18.0 (14.0, 18.0)	16.0 (14.0, 18.0)	16.0 (16.0, 20.0)	18.0 (16.0, 20.0)	<0.001
CL, (%)	9 (6.3)	15 (4.0)	17 (7.4)	76 (9.1)	0.004
Collateral circulation, (%)	2 (1.4)	10 (2.7)	12 (5.2)	46 (5.5)	0.006
LMPCI, (%)	8 (5.6)	8 (2.1)	2 (0.9)	6 (0.7)	0.001
LADPCI, (%)	66 (46.2)	194 (51.5)	122 (53.0)	416 (50.1)	0.833
LCXPCI, (%)	37 (25.9)	85 (22.5)	45 (19.6)	183 (22.0)	0.411
RCAPCI, (%)	66 (46.2)	140 (37.1)	76 (33.0)	319 (38.4)	0.388
Number of stents, M (Q1, Q3)	2.0 (1.0, 2.00)	1.0 (1.0, 2.0)	1.0 (1.0, 2.0)	1.0 (1.0, 2.0)	0.176
Number of Balloon, M (Q1, Q3)	2.0 (2.0, 3.0)	2.0 (2.0, 3.0)	2.0 (2.0, 3.0)	2.0 (2.0, 3.0)	0.002
Imported balloon, (%)	108 (75.5)	271 (71.9)	86 (37.4)	249 (30.0)	<0.001
Imported sent, (%)	6 (4.2)	18 (4.8)	11 (4.8)	65 (7.8)	0.065
LM/3VD, (%)	90 (62.9)	176 (46.7)	71 (30.9)	141 (17.0)	<0.001
Multivessel disease, (%)	121 (84.6)	298 (79.0)	138 (60.0)	354 (42.6)	<0.001
Shock, (%)	5 (3.5)	3 (0.8)	8 (3.5)	6 (0.7)	0.904
Bleeding, (%)	0 (0.0)	1 (0.3)	2 (0.9)	3 (0.4)	0.680
BMI, M (Q1, Q3)	24.5 (22.9, 27.0)	24.8 (22.7, 27.1)	24.2 (22.9, 26.2)	24.2 (22.9, 26.1)	0.022
LDH, U/L, M (Q1, Q3)	268.0 (184.5, 560.0)	236.0 (177.0, 388.0)	222.0 (181.5, 377.5)	213.0 (179.0, 296.5)	0.003
CK, U/L, M (Q1, Q3)	179.0 (89.0, 962.0)	173.0 (89.0, 731.0)	115.5 (77.4, 324.6)	125.0 (78.0, 293.0)	<0.001
CKMB, U/L, M (Q1, Q3)	28.0 (16.0, 95.9)	23.0 (14.0, 71.0)	10.5 (5.0, 34.7)	10.0 (5.8, 25.0)	<0.001
cTnI, ng/ml, M (Q1, Q3)	0.21 (0.1, 5.7)	0.16 (0.1, 5.1)	0.11 (0.0, 4.8)	0.07 (0.0, 1.6)	<0.001
TC, mmol/L, M (Q1, Q3)	4.9 (4.0, 6.0)	4.6 (3.9, 5.5)	5.0 (4.0, 6.0)	4.6 (3.9, 5.4)	0.489
TG, mmol/L, M (Q1, Q3)	1.6 (1.1, 2.2)	1.6 (1.1, 2.3)	1.4 (1.0, 2.0)	1.4 (0.9, 2.1)	<0.001
LDL-C, mmol/L, M (Q1, Q3)	3.1 (2.5, 3.9)	2.8 (2.2, 3.5)	3.0 (2.1, 3.8)	2.7 (2.1, 3.4)	0.052
HDL-C, mmol/L, M (Q1, Q3)	1.3 (1.0, 1.6)	1.3 (1.0, 1.6)	1.1 (1.0, 1.4)	1.1 (1.0, 1.3)	<0.001
BUN, mmol/L, M (Q1, Q3)	5.7 (4.6, 7.2)	5.1 (4.2, 6.2)	6.1 (5.0, 7.5)	5.5 (4.5, 6.6)	<0.001
Crea, μmol/L, M (Q1, Q3)	67.0 (55.0, 85.0)	66.0 (55.0, 78.0)	70.0 (58.0, 84.0)	64.0 (54.9, 75.0)	0.280
UA, μmol/L, M (Q1, Q3)	290.0 (239.0, 360.5)	273.0 (221.0, 341.0)	313.6 (258.0, 378.6)	302.2 (242.9, 372.3)	<0.001
eGFR, ml/min/1.72 m^2^, M (Q1, Q3)	83.5 (63.8, 104.7)	97.0 (78.8, 119.3)	81.0 (60.4, 99.0)	96.1 (79.1, 118.4)	0.754
WBC, 10×^9^/L, M (Q1, Q3)	7.9 (6.6, 10.7)	7.6 (6.1, 9.7)	7.4 (6.0, 9.6)	7.4 (6.1, 9.5)	0.090
LYMPH, 10×^9^/L, M (Q1, Q3)	1.6 (1.2, 2.0)	1.6 (1.2, 2.1)	1.6 (1.2, 2.1)	1.7 (1.3, 2.2)	0.001
LYMPH%, M (Q1, Q3)	18.7 (12.8, 27.2)	22.3 (15.2, 28.6)	23.0 (17.6, 29.6)	23.9 (17.1, 30.5)	<0.001
NEUT, 10×^9^/L, M (Q1, Q3)	5.5 (3.9, 8.6)	5.0 (3.9, 7.1)	4.8 (3.6, 6.5)	4.8 (3.7, 6.8)	0.005
NEUT%, M (Q1, Q3)	71.6 (62.2, 79.3)	68.1 (60.5, 75.8)	67.2 (61.1, 73.8)	65.9 (58.5, 74.0)	<0.001
NEUT/LYMPH, M (Q1, Q3)	3.8 (2.2, 6.4)	3.1 (2.1, 5.3)	2.9 (2.1, 4.1)	2.8 (1.9, 4.3)	<0.001
RBC, 10×^9^/L, M (Q1, Q3)	4.4 (4.1, 4.8)	4.5 (4.2, 4.8)	4.5 (4.1, 4.9)	4.6 (4.3, 5.0)	0.003
PLT, 10×^9^/L, M (Q1, Q3)	202.0 (174.5, 248.5)	206.0 (175.0, 249.0)	197.5 (160.3, 248.8)	206.0 (175.0, 244.0)	0.502
MPV, fL, M (Q1, Q3)	9.9 (7.9, 10.8)	10.0 (8.9, 10.7)	10.2 (9.6, 10.9)	10.2 (9.6, 10.9)	<0.001
Hb, g/L, M (Q1, Q3)	136.0 (125.0, 149.0)	140.0 (128.0, 150.0)	134.5 (126.0, 149.0)	141.0 (131.0, 152.0)	0.027
APTT, s, M (Q1, Q3)	27.3 (24.2, 30.5)	26.8 (24.1, 30.0)	29.4 (25.7, 33.3)	28.5 (25.5, 32.6)	<0.001
FPG, mmol/L, M (Q1, Q3)	6.4 (5.4, 8.6)	6.4 (5.4, 8.1)	6.3 (5.4, 7.9)	6.4 (5.5, 8.2)	0.920
ALT, U/L, M (Q1 , Q3)	24.0 (15.0, 40.5)	24.0 (16.0, 38.0)	25.0 (17.0, 41.8)	29.0 (18.0, 45.0)	<0.001
AST, U/L, M (Q1, Q3)	25.0 (18.0, 71.5)	26.0 (19.0, 48.0)	25.0 (20.3, 49.8)	27.0 (20.0, 51.0)	0.241
GGT, U/L, M (Q1, Q3)	28.0 (17.0, 42.0)	26.0 (18.0, 42.0)	25.0 (17.0, 40.9)	26.0 (18.0, 38.0)	0.356
ALB, g/L, M (Q1, Q3)	40.1 (37.3, 42.0)	40.6 (38.5, 42.6)	41.0 (38.0, 43.6)	41.8 (39.2, 44.6)	<0.001
LVEF, %, M (Q1, Q3)	55.0 (50.0, 61.0)	59.0 (55.0, 63.0)	57.0 (50.0, 60.0)	59.0 (55.0, 62.0)	0.672
LAD, mm, M (Q1, Q3)	35.0 (32.0, 40.0)	33.00 (30.0, 37.0)	34.0 (31.0, 38.0)	33.0 (30.0, 36.0)	<0.001
LVEDD, mm, M (Q1, Q3)	48.0 (44.0, 51.0)	46.0 (44.0, 50.0)	47.0 (44.0, 51.0)	47.0 (44.0, 50.0)	0.020
Medications, (%)					
Aspirin	138 (96.5)	370 (98.1)	221 (96.1)	808 (97.2)	0.521
P2Y_12_ inhibitor	137 (95.8)	371 (98.4)	218 (94.8)	800 (96.3)	0.102
LMWH	132 (92.3)	356 (94.4)	196 (85.2)	667 (80.3)	<0.001
Statins	139 (97.2)	370 (98.1)	218 (94.8)	784 (94.3)	0.003
β-blockers	97 (67.8)	232 (61.5)	124 (53.9)	488 (58.7)	0.038
RAAS Inhibitors	84 (58.7)	215 (57.0)	125 (54.3)	451 (54.3)	0.249
CCB	97 (67.8)	256 (67.9)	114 (49.6)	486 (58.5)	<0.001
Spironolactone	36 (25.2)	49 (13.0)	36 (15.7)	102 (12.3)	0.828
PPI	55 (38.5)	124 (32.9)	137 (59.6)	493 (59.3)	0.045
InhAPmed	96 (67.1)	257 (68.2)	147 (63.9)	572 (68.8)	1.000
TCM	86 (60.1)	240 (63.7)	184 (80.0)	637 (76.7)	<0.001

MI, myocardial infarction; TA, thrombus aspiration; MDP, maximum dilation pressure; CL, culprit lesions; LMPCI, PCI of left main coronary artery; LADPCI, PCI of left anterior descending coronary artery; LCXPCI, PCI of left circumflex coronary artery; RCAPCI, PCI of right coronary artery; NODAP, new-onset diabetes after PCI; RAAS, renin-angiotensin-aldosterone system; β-blockers, beta-adrenergic receptor blockers; LDH, lactate dehydrogenase; CK, creatine kinase; CKMB,CK isoenzyme; cTnI, cardiac troponin I;TC, total cholesterol; TG, triglycerides; LDL-C, low-density lipoprotein; HDL-C, high density lipoprotein; BUN, blood urea nitrogen; UA, uric acid; eGFR, estimated glomerular filtration rat; WBC, white blood cell; LYMPH, lymphocyte; NEUT, neutrophil granulocyte; RBC, red blood cell; PLT, blood platelet; MPV, mean platelet volume, Hb, hemoglobin; APTT, activated partial thromboplastin time; FPG, fasting plasma glucose (FPG); ALT, alanine aminotransferase; AST, aspartate aminotransferase; GGT, glutamyl transpeptidase; ALB, albumin; LVEF, left ventricular ejection fraction; LAD, left atrial diameter; LVEDD, left ventricular end diastolic dimension; LMWH, low molecular weight heparin; CCB, calcium channel blockers; PPI, proton pump inhibitor; TCM, traditional Chinese medicine.

Through telephone (to the patients themselves or their relatives), patients were followed up in the first, second, and third years post-PCI, with no further follow-up if a death event was recorded. During follow-up, medication use, daily behavioral habits, and outcome events, including all-cause death, target lesion revascularization, recurrent myocardial infarction, stroke, heart failure, rehospitalization for cardiac reasons, and major bleeding events, were all recorded. The general condition of the patients and the medications taken during the follow-up period are depicted in Supplementary Table S3.

### 2.4. Statistical analysis

Normally distributed variables were summarized as means and standard deviations, medians and interquartile ranges represented skewed distributional data, and frequencies or proportions were used to describe categorical variables. The *t*-test, the rank-sum test, and the χ^2^ test were applied to compare continuous variables of normal distribution, continuous variables of non-normal distribution, and categorical variables, respectively.

As candidate predictors, 97 clinical features with at least 70% data completeness were evaluated. For the missing values, multiple imputation was performed using random forest. The most useful predictors were filtered using the Least Absolute Shrinkage and Selection Operator (LASSO) regression, which was additionally augmented with 10-fold cross-validation for internal validation, incorporating penalty parameter tuning based on minimum criteria and 1 standard error (SE) of the minimum criteria. The detailed description of the LASSO model is shown in Section 1.3 of the Supplementary Material. The most predictive covariates were selected by lambda.1se. The predictor factors discovered by the LASSO regression analysis were incorporated using the multivariate logistic regression model. Subsequently, the predictor variables that consistently achieved statistical significance were used to generate the risk score and were represented by the nomogram.

Furthermore, Restricted Cubic Splines (RCS) were used to analyze the association of continuous variables with MACEs incidence among ACS patients post-PCI. The reference values (OR = 1) were set at the 10th percentiles, and four knots were placed at the 5th, 35th, 65th, and 95th percentiles of the distribution, respectively. Following that, continuous variables that reported non-linear association were transformed based on RCS and clinical experience to develop an improved predictive model. All statistical analyses were performed using SPSS (version 26.0) and R software (Version R-4.1.3).

## 3. Results

### 3.1. Characteristics of patients and outcome

After excluding those who were lost to follow-up or had missing data, the study comprised 1,581 ACS patients who underwent PCI. The training and validation sets included 520 and 1,061 patients, respectively. The baseline and follow-up characteristics of patients in the training and validation sets are respectively displayed in Table 1 and Supplementary Table S3. During follow-up, MACEs were detected in 143 (27.5%) but not in 377 training data set instances and in 230 (21.7%) but not in 831 validation set cases. The distribution of clinical outcomes in ACS patients with different subtypes of coronary artery disease in the training and external validation sets is shown in Supplementary Tables S1,S2.

### 3.2. Predictor selection

The LASSO regression included 97 factors evaluated at admission and follow-up (Table 1 and Supplementary Table S3). Twenty-one variables (Age, Killip Functional Classification III-IV (Killip III-IV), Hypertension, dyslipidemia, Hyperhomocysteinemia (HHcy), Heart Failure (HF), Chronotropic Incompetence (CI), Pulmonary Heart Disease (PHD), Number of stents, Multivessel disease, New-Onset Diabetes after PCI (NODAP), Aspartate Aminotransferase (AST), Low-Density Lipoprotein Cholesterol (LDL-C), estimated Glomerular Filtration Rate (eGFR), Total Cholesterol (TC), Blood Urea Nitrogen (BUN), Absolute Neutrophil Count (ANC), Left Ventricular Ejection Fraction (LVEF), Left Atrial Diameter (LAD), Maximum Dilation Pressure (MDP), P2Y12 Receptor Antagonist (P2Y12-RA) use after PCI were found to continue to be significant predictors of MACEs (Supplementary Figure S3).

### 3.3. Association between continuous variables and predicted outcomes

The correlation between continuous variables selected through LASSO regression and the anticipated outcome was analyzed before developing the prognostic program. We employed restricted cubic splines (RCS) to graphically represent non-linear associations (Supplementary Table S4). In both the training and external validation sets, the variable LVEF showed a non-linear association with the predicted outcome MACEs (Supplementary Figure S4).

### 3.4. Development of the multivariate prognostic nomogram

**Figure 1:**
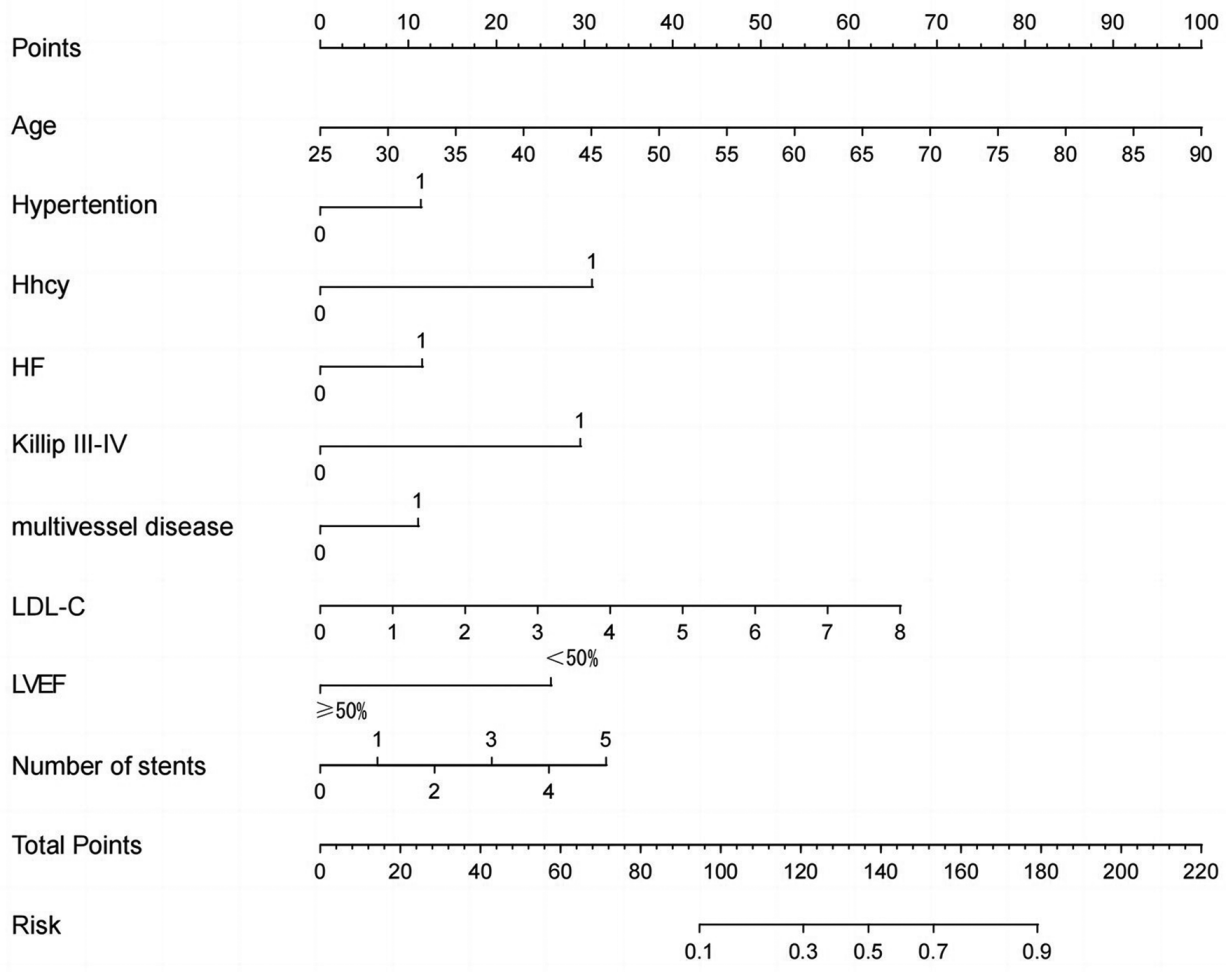
Nomogram used for predicting MACEs after PCI in ACS patients.

According to the univariate and multivariate logistic regression analysis (*P* < 0.05) (Table 2), 9 out of 21 prospective clinical factors were independently statistically significant predictors of MACEs in the training set and were incorporated in the prognostic nomogram (Figure 1). These variables included Age (OR, 1.08; 95% CI, 1.06–1.11; *P* < 0.001), Killip III-IV (OR, 4.65; 95% CI, 1.43–15.14; *P* = 0.011), Hypertension (OR, 1.81; 95% CI, 1.11–2.97; *P* = 0.018), HHcy (OR, 4.99; 95% CI, 1.46–17.04; *P* = 0.010), HF (OR, 1.83; 95% CI, 1.02–3.26; *P* = 0.042), Number of stents (OR, 1.40; 95% CI, 1.09–1.80; *P* = 0.008), Multivessel disease (OR, 1.78; 95% CI, 1.13–2.83; *P* = 0.014), LDLC (OR, 1.54; 95% CI, 1.22–1.93; *P* < 0.001), and LVEF (OR, 0.26; 95% CI, 0.13–0.49; *P* < 0.001). A straight line drawn from the point axis upward connected each predictor in a prognostic nomogram to a specific point. The “Total Points” axis was used to display the sum of scores for each variable. The plotted “Total Points” axis was subsequently connected directly to the probability axis by a vertical line to determine the probability of MACEs.

**Table 2:** Univariate and multivariable logistics regression analysis of predictive variables in the training set.

Variable	Univariate		Multivariate	
	OR (95% CI)	*P*-value	OR (95% CI)	*P*-value
Age	1.08 (1.06, 1.11)	<0.001	1.08 (1.06, 1.11)	<0.001
Hypertension	1.57 (1.03, 2.40)	0.034	1.81 (1.11, 2.97)	0.018
HHcy	4.65 (1.66, 13.04)	0.003	4.99 (1.46, 17.04)	0.010
HF	2.45 (1.50, 4.02)	<0.001	1.83 (1.02, 3.26)	0.042
Number of stents	1.39 (1.12, 1.72)	0.003	1.40 (1.09, 1.80)	0.008
Killip III-IV	5.66 (2.08, 15.4)	<0.001	4.65 (1.43, 15.14)	0.011
multivessel disease	1.94 (1.31, 2.88)	0.001	1.78 (1.13, 2.83)	0.014
LDL-C	1.36 (1.11, 1.65)	0.003	1.54 (1.22, 1.93)	<0.001
LVEF ≥ 50%*	0.24 (0.13, 0.42)	<0.001	0.26 (0.13, 0.49)	<0.001

* According to the RCS combined with clinical experience, the LVEF was considered as < 50% and ≥ 50%.

### 3.5. Performance of the prognostic nomogram

The Area Under the ROC Curve (AUC) for the model in the training set was 0.79 (95% CI 0.75–0.84), which showed excellent discrimination compared to the GRACE score (Supplementary Figure S5a). The model's cutoff value was 0.30, and based on the optimal cutoff points, the sensitivity and specificity were 76.90% and 69.50%, respectively. The calibration plot for the risk of MACEs demonstrated a substantial agreement between nomogram prediction and actual observation (Supplementary Figure S6a).

### 3.6. Validation of the prognostic nomogram

In the validation set, the nomogram also demonstrated superior ability in predicting the risk of MACEs after PCI compared to the GRACE score. According to the ROC curve, the AUC value of the model was 0.75 (95% CI, 0.71–0.78) (Supplementary Figure S5b), and with these results, the validation set likewise confirmed the nomogram's favorable calibration (Supplementary Figure S6b).

### 3.7. Clinical utility

Furthermore, the clinical validity of the nomogram regarding its clinical utility was evaluated using a Decision Curve Analysis (DCA) and Clinical Impact Curve (CIC) (Supplementary Figures S7,S8). The DCA showed that the decision curve of the model is located above the None and All lines when the threshold probability of MACEs post-PCI in ACS patients was between 0.08 and 0.98, implying that, within that reasonable level, the prediction model delivered a significant net benefit. In other words, using the prognostic nomogram to predict postoperative MACEs in ACS patients was more favorable than the all-patient tactics or the no-patient tactics. In addition, when compared to the GRACE score, the prognostic nomogram showed a larger range of thresholds as well as greater net benefit over much of the threshold range, indicating that it is a substantially superior scoring system. The CIC shows the clinical validity of the prognostic nomogram. When the threshold probability was greater than the 65% predictive score probability value, the prognostic nomogram determined that the population at high risk for MACEs was highly matched to the population that actually experienced MACEs, confirming the high clinical effectiveness of the prognostic nomogram. In conclusion, these findings supported the clinical applicability and accuracy of the nomogram in predicting the probability of MACEs in ACS patients post-PCI.

## 4. Discussion

Based on the increased CHD incidence, its health and economic burdens have increased considerably (20). Although the prognosis of CHD patients has improved due to expanded PCI use, estimating the long-term risk of MACEs among ACS patients post-PCI is frequently still required due to residual cardiovascular risk (21, 22). Atherosclerosis is a chronic disease process that dynamically develops in the cardiovascular setting (23). Several factors related to inflammation, the immune system, and metabolic disorders arising from genetic, environmental, and behavioral drivers accelerate the progression of atherosclerosis, which subsequently contributes to the development of ACS (24–29). Thus, rather than any single factor, the onset and progression of ACS and its residual risk are influenced by the interaction of multisystemic factors.

Several risk and prognosis assessment tools for cardiovascular diseases have been developed to guide clinical practice by identifying individuals at increased risk for MACEs across various populations (30–33). These tools, along with their corresponding scores, can inform clinical decisions for secondary prevention by identifying high-risk patients who might require ancillary clinical assistance and resources. However, in addition to some of them requiring complex completion procedures, the clinical validity of these scores is limited. Furthermore, different studies have revealed discrepancies in prognostic judgments for the above scores. For example, GRACE, TIMI, Zwolle, and CADILLAC scores were employed to analyze the 5-year prognosis of STEMI patients post-PCI. Kozieradzka et al. found that for predicting all-cause mortality, the CADILLAC model had the lowest discrimination (14). After comparing the prognostic accuracy of six scoring models for three-year mortality in STEMI patients, Jarkovsky et al. discovered that longer follow-up periods could best be predicted by GRACE (15). Scruth et al., on the other hand, concluded that CADILLAC and TIMI scores were better predictors of major cardiac events at one year (16).

Several currently popular models used to evaluate the risk of cardiovascular disease may not have been adequately optimized for long-term prognosis and the consistent use of scoring by clinicians over time. These models exhibit varying outcomes and pose significant decision-making risks in real-world clinical settings, particularly among patients with acute coronary syndrome undergoing percutaneous coronary intervention at county-level hospitals. Consequently, the present study aimed to integrate routinely available characteristics during PCI treatment and follow-up in multicenter county-level hospitals to develop a nomogram for predicting the occurrence of MACEs after PCI in ACS patients. The population included in this study was ACS patients treated with PCI from 2018 to 2019, which is closer to the real world than previously registered scores, and the nomogram modelling demonstrated more accurate predictive efficacy than the conventional Grace scoring system. Consequently, our nomogram can serve as a valuable adjunct to existing risk scores.

Previous research has linked the clinical profile of ACS patients at admission to prognosis, with age, cardiac insufficiency, and blood pressure clinically recognized as independent markers of poor prognosis following PCI in ACS patients (34–53).

In addition to being the most commonly used measure of left ventricular systolic function, Left Ventricular Ejection Fraction (LVEF), as measured by transthoracic echocardiography, is one of the strongest predictors of MACEs occurrence post-PCI in patients with coronary artery disease. At admission, the LVEF determines the extent of the decline in the left ventricle's systolic function. Individuals are more likely to experience severe cardiovascular endpoint events due to the long-term decline in cardiac output caused by myocardial infarction. According to the American Society of Echocardiography and the European Society of Cardiovascular Imaging, specific thresholds of 52% and 54% for men and women, respectively, define an increased risk of left ventricular dysfunction and early death (54). Tajstra et al. demonstrated that in ischemic heart failure (LVEF ≤ 35%), patients with chronic, completely occlusive lesions had a worse long-term prognosis (55). An observational study of 230,464 cases from the British Cardiovascular Intervention Society angioplasty database revealed that compared to patients with preserved ejection fraction (50%), patients with moderately impaired LV ejection fraction (30%–49%) had a threefold increase in 30-day post-PCI mortality (56). As revealed by a study summarizing five randomized clinical trials, patients with reduced ejection fraction (<40%) or median ejection fraction (40%–49%) had an increased risk of all-cause mortality, cardiac death, and a composite risk of cardiac death in the context of coronary artery disease treated with clinically indicated PCI (57). In contrast, recent cohort studies have shown inconsistent findings, with ejection fraction below 60% or over 65% being associated with an increased mortality risk. These findings contradict the previous consensus that associated an increased mortality risk only with severely reduced ejection fraction (58). Furthermore, an Australian study discovered that at higher ejection fraction levels, women had a greater risk of death (59). Since these studies were conducted on the general population undergoing echocardiography, the association between LVEF and adverse outcomes in coronary artery disease patients following PCI remains unclear. Herein, we attempted to examine the connection between LVEF and the risk of MACEs in ACS patients undergoing PCI in a large observational cohort of county-level multicenter hospitals. Using restricted cubic splines, we evaluated a non-linear relationship between LVEF and adverse cardiovascular events. We discovered that compared with patients with preserved ejection fraction (>50%), patients with reduced ejection fraction (≤50%) had a higher incidence of post-PCI MACEs. This result is consistent with the findings of a prior study, which indicated that when LVEF was evaluated using cardiac magnetic resonance, the TIMI risk score had an improved capacity to predict all-cause mortality, reinfarction, and new-onset congestive heart failure within a year following infarction (60). In addition, to minimize the confounding effect of different assessment times, we completed all echocardiographic assessments during hospitalization in our study. Images were not evaluated by a central laboratory in our study, which may introduce subjectivity in the interpretation of echocardiographic data. However, our goal was to reflect true clinical practice, in which ultrasound assessments are performed by a variety of health care providers.

Additionally, the multivessel disease was found to be an independent predictor of MACEs in patients post-PCI (60, 61). An increased number of diseased vessels indicates more extensive and complex coronary lesions, necessitating more stents, balloons, and other interventional devices used during PCI, which substantially increase the risk of damage to coronary vessels and cardiomyocytes, and in turn leading to thrombosis and microcirculatory disorders, consequently increasing the risk of post-PCI adverse events (42, 53, 62, 63).

The other independent factor in predicting adverse events in ACS patients was serum homocysteine (Hcy) levels. Homocysteine-induced endothelial dysfunction, lipid peroxidation, and peroxisome proliferator-activated receptor inactivation may enhance smooth muscle cell proliferation, extracellular matrix production, and MACE risk after PCI (64). Secondary prevention using folic acid and other B vitamins failed to prevent or minimize cardiovascular events in some randomized controlled studies, despite a connection between raised plasma HCY levels and late cardiac events and poor prognosis in CAD patients (65–70). Thus, more clinical trials are needed to demonstrate the predictive usefulness of HCY levels on coronary atherosclerosis development, late stent failure, and long term clinical outcomes.

Following the adjustment for other clinical traits in the study's participants, we also observed that LDL-C levels remained associated with the risk of adverse events in PCI recipients. This finding stresses the importance of appropriate risk reduction methods as it underlines the large residual risk in these patients despite successful revascularization. Furthermore, a multicenter study investigating the association between LDL-C and long-term cardiovascular events post-PCI linked higher LDL-C levels with an increased risk of late cardiovascular events (71). Therefore, prompt initiation of intensive statin therapy may provide early clinical benefit after ACS, and long-term adherence to optimal lipid-lowering therapy may effectively reduce long-term cardiovascular events post-PCI (65).

Notably, due to urbanization, significant geographical disparities were observed in the course and prognosis of ACS patients in developing nations, with provincial hospital patients being younger and having lower fatality rates than those treated in district hospitals (72, 73). Furthermore, the Prospective Urban Rural Epidemiologic (PURE) study revealed that underprivileged populations in low-income countries faced challenges regarding access to primary and secondary prevention (5). As a quantitative tool for evaluating clinical risk and benefit, our nomogram has demonstrated strong predictive performance, enabling clinicians to promptly identify patients in need of active attention and frequent follow-up. This allows for early prognostic risk assessment, the development of personalized treatment plans, and the establishment of follow-up schedules to effectively guide patient management. Early detection and diagnosis of MACEs after PCI in patients with ACS can be achieved by predicting their increased risk during follow-up. This can be done through intensive electrocardiogram monitoring in community healthcare units. Implementing this approach can help ensure consistent and standardized preventive treatment for high-risk populations in remote areas. Additionally, it can address the challenges of limited access to hospitals and high hospitalization costs for low-income populations.

## 5. Limitations

First, this study created a clinical prediction model by examining independent risk factors for long-term MACEs in PCI patients. Thus, the chosen indicators primarily comprise those commonly employed in clinical contexts.

Second, the study exclusively assessed the prognostic accuracy of the predictive nomogram in ACS patients undergoing PCI. More research is needed to determine whether the predictive nomogram exerts a similar clinical effect on ACS patients undergoing alternative therapies, such as coronary artery bypass graft.

Third, due to the low reliability of telephone follow-up, the study has not distinguished between cardiovascular and non-cardiovascular as well as cancer-specific causes of mortality.

Fourth, since the study was conducted across multiple centers, there were variations in surgical equipment and physician experience. As a result, the PCI outcomes were inevitably impacted, potentially affecting the prognosis of patients. Therefore, the findings of this investigation could be compromised.

Fifth, this study has not directly compared patient outcomes with those of patients treated at other hospital levels. Therefore, the findings are exclusive to the county-level hospital environment.

## 6. Conclusion

In summary, our nomogram aims to provide novel perspectives for county-level post-PCI rehabilitation programs in LMIC, and consequently lower the incidence of severe adverse cardiovascular events in post-PCI patients.

## Data Availability

The raw data supporting the conclusions of this article will be made available by the authors, without undue reservation.
