# Supplementary material for: Predicting long-term prognosis after percutaneous coronary intervention in patients with acute coronary syndromes: a prospective nested case-control analysis for county-level health services
Source: Front Cardiovasc Med. 2023 Dec 4;10:1297527. doi: 10.3389/fcvm.2023.1297527 (PMC10725923; doi:10.3389/fcvm.2023.1297527)
Supplement: Supplementary file 1 [file Table1.docx]

Supplementary Material

Predicting long-term prognosis after percutaneous coronary intervention in patients with acute coronary syndromes: a prospective nested case-control analysis for county-level health services

# SUPPLEMENTARY EXPANDED METHODS

## The diagnostic criteria for acute coronary syndromes (ACS).

## Figure S1 ACS patient occupations and payment methods distribution among centers.

## The detailed description of the Least absolute shrinkage and selection operator (LASSO) Model.

# SUPPLEMENTARY RESULTS

## Table S1 Distribution of clinical outcomes in ACS patients with different subtypes of coronary artery disease in the training set.

## Table S2 Distribution of clinical outcomes in ACS patients with different subtypes of coronary artery disease in the external validation set.

## Table S3 Characteristics of ACS patients during follow-up.

## Table S4 Results of Linear hypothesis test with the RCS function.

## Figure S2 Study flow diagram.

## Figure S3 Feature selection using the LASSO binary logistic regression model.

## Figure S4 Association of LVEF with risk of MACEs.

## Figure S5 ROC curve of the nomogram for predicting the risk of MACEs after PCI in ACS patients.

## Figure S6 Calibration curve of the nomogram for the training set and the validation set.

## Figure S7 Decision curve analysis (DCA) of the nomogram.

## Figure S8 Clinical Impact Curve (CIC) of the nomogram.

# SUPPLEMENTARY REFERENCES

**SUPPLEMENTARY EXPANDED METHODS**

**The diagnostic criteria for acute coronary syndromes (ACS)**

**These diagnostic criteria were according to the previously published definitions and literatures, together with the consensus of Chinese Society of Cardiology of Chinese Medical Association(1-3).**

|  | **DEFINITIONS** |
| --- | --- |
| **STEMI** | Dynamic changes in serum troponin levels with at least 1 test suggesting a level greater than the 99th upper limit of normal with clinical evidence of acute myocardial ischaemia:   1. acute persistent ischaemic precordial pain; 2. new ischaemic changes on ECG, such as ST-segment arch dorsal elevation and pathological Q waves; 3. new myocardial infarction or abnormal ventricular segmental motion on ultrasonography; 4. coronary angiography or intracavitary imaging suggesting coronary artery disease. Internal imaging suggests the presence of a thrombus in the coronary artery. |
| **NSTEMI** | Dynamic changes in serum troponin levels with at least 1 test suggesting a level greater than the 99th upper limit of normal with clinical evidence of acute myocardial ischaemia:   1. clinical signs of acute ischaemic chest pain; 2. new ischaemic changes on ECG such as ST-segment downshift, transient ST-segment elevation and T-wave changes; 3. abnormal ventricular wall segmental motion on echocardiography; 4. coronary angiography or intracavitary imaging confirming coronary artery thrombosis. |
| **UA** | One of the following criteria is necessary:   1. Angina that occurred at rest and was prolonged, usually lasting ≥ 10 min; 2. New-onset angina of at least Canadian Cardiovascular Society (CCS) classification III severity; 3. Recent acceleration of angina reflected by an increase in severity of at least 1 CCS class to at least CCS class III. 4. The patient must also not have any biochemical evidence (cardiac troponin) of myocardial necrosis. 5. Confirmed ischemic electrocardiographic (ECG) changes is not necessary if the local cardiologists and an independent Eligibility Committee think a diagnosis of UA is established. |


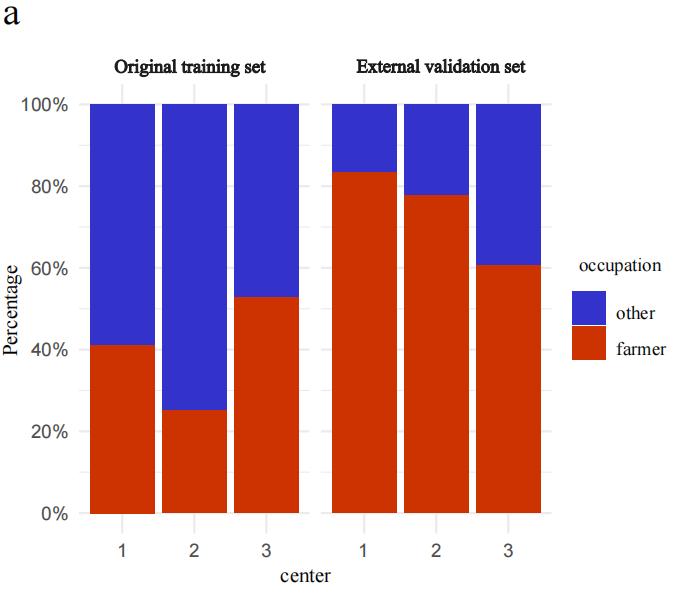

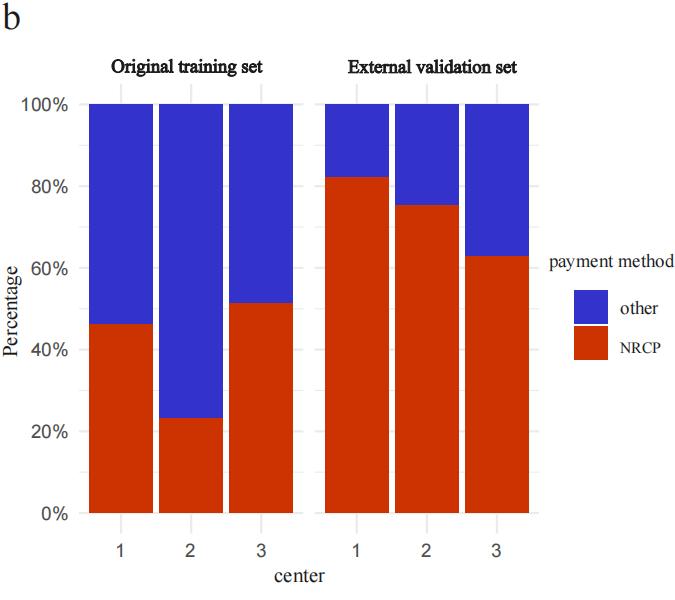


**Figure S1** ACS patient occupations and payment methods distribution among centers.

(**a** shows ACS patient occupations distribution among centers in the training set and external validation set; **b** shows ACS patient payment method distribution among centers in the training set and external validation set; NRCP:New Rural Cooperative Medical Plan)

**The detailed description of the Least absolute shrinkage and selection operator (LASSO) Model.**

The rationale of LASSO is to shrink the regression coefficients of each independent variable by constructing a penalty function. An appropriate penalty parameter lambda can shrink some of the coefficients, minimizing the sum of squared residuals, and even render some coefficients zero, thus eliminating them from the model. As a result, a model with good explanatory power and conciseness is produced.(4-6).This provides the conditions for us to extract variables with a higher correlation to adverse cardiovascular events post-PCI in ACS patients for further model construction

We chose to further analyze the original cohort sample using the LASSO regression method. This was accomplished using the glmnet package in R (Version R-4.1.3), with family="binomial" defined. The selection of the penalty parameter lambda was conducted using K-fold cross-validation, where we set K to 10, meaning 10-fold cross-validation was used to select lambda. The original cohort sample was randomly divided into 10 groups, with each iteration using 9 groups for training and the remaining one for validation. This process was repeated, and the mean square error (MSE) was used to determine the final parameter lambda. The results from the glmnet package indicated that at the minimum MSE, lambda.min = 0.017, and at the maximum value within one standard error of the minimum MSE, lambda.1se = 0.033, as shown in Figure 1b. In the cross-validation results, the two lambda parameters—one at the minimum MSE and the other at the maximum value within one standard error of the minimum MSE—the former, due to a relatively smaller degree of compression on regression coefficients, might not fully resolve the issue of overfitting and may not fully meet the objectives of LASSO modeling. Therefore, we chose lambda as the maximum value within one standard error of the minimum MSE to ensure the model performs well with the fewest number of independent variables. The results of variable selection showed that as the lambda value increased, the penalty strength also increased, shrinking the regression coefficients of the 76 variables in the model to 0 until all were 0 (Figure 1a). At a lambda value of 0.033, the model retained 21 non-zero coefficients, indicating that 21 independent variables were preserved.

**SUPPLEMENTARY RESULTS**

**Table S1**: **Distribution of clinical outcomes in patients with different subtypes of coronary artery disease in the training set**

**Table S2 Distribution of clinical outcomes in patients with different subtypes of coronary artery disease in the external validation set**

| ACS typing | N | MACE | | | | | | | | | | | | | | |
| --- | --- | --- | --- | --- | --- | --- | --- | --- | --- | --- | --- | --- | --- | --- | --- | --- |
|  |  | stroke | |  | heart failure | |  | target lesion revascularization | |  | recurrent myocardial infarction | |  | all-cause death | |  |
|  |  | **N** | **P()%** |  | **N** | **P()%** |  | **N** | **P()%** |  | **N** | **P()%** |  | **N** | **P()%** |  |
| STEMI | 229 | 5 | 2.2% |  | 36 | 15.7% |  | 12 | 5.2% |  | 40 | 17.5% |  | 18 | 7.9% |  |
| NSTEMI | 119 | 2 | 1.7% |  | 15 | 12.6% |  | 2 | 1.7% |  | 9 | 7.6% |  | 9 | 7.6% |  |
| UA | 172 | 5 | 2.9% |  | 20 | 11.6% |  | 11 | 6.4% |  | 20 | 14.0% |  | 7 | 4.1% |  |
| Total | 520 | 12 | 2.3% |  | 71 | 13.7% |  | 25 | 4.8% |  | 73 | 14.0% |  | 34 | 6.5% |  |

| ACS typing | N | MACE | | | | | | | | | | | | | | |
| --- | --- | --- | --- | --- | --- | --- | --- | --- | --- | --- | --- | --- | --- | --- | --- | --- |
|  |  | stroke | |  | heart failure | |  | target lesion revascularization | |  | recurrent myocardial infarction | |  | all-cause death | |  |
|  |  | **N** | **P()%** |  | **N** | **P()%** |  | **N** | **P()%** |  | **N** | **P()%** |  | **N** | **P()%** |  |
| STEMI | 354 | 11 | 3.1% |  | 37 | 10.5% |  | 11 | 3.1% |  | 29 | 8.2% |  | 21 | 5.9% |  |
| NSTEMI | 328 | 8 | 2.4% |  | 28 | 8.5% |  | 16 | 4.9% |  | 39 | 11.9% |  | 19 | 5.8% |  |
| UA | 379 | 8 | 2.1% |  | 25 | 6.6% |  | 17 | 4.5% |  | 37 | 9.8% |  | 14 | 3.7% |  |
| Total | 1061 | 27 | 2.5% |  | 90 | 8.5% |  | 44 | 4.1% |  | 105 | 9.9% |  | 54 | 5.1% |  |

**Table S3**: **Characteristics of ACS patients during follow-up**

| Variable | Training set (N=520) | | External validation set (N=1061) | | *P*-value |
| --- | --- | --- | --- | --- | --- |
|  | MACEs (N=143) Controls (N=377) | | MACEs (N=230) Controls (N=831) | |  |
| Smoking after PCI, (%) |  |  |  |  | 0.006 |
| None | 90 (62.9) | 233 (61.8) | 162 (70.4) | 572 (68.8) |  |
| Quitting | 26 (18.2) | 78 (20.7) | 27 (11.7) | 129 (15.5) |  |
| Reduction | 17 (11.9) | 44 (11.7) | 26 (11.3) | 67 (8.1) |  |
| Smoking | 10 (7.0) | 22 (5.8) | 15 (6.5) | 63 (7.6) |  |
| Blood pressure control, (%) |  |  |  |  | 0.016 |
| Normal | 108 (75.5) | 303 (80.4) | 189 (82.2) | 711 (85.6) |  |
| High | 26 (18.2) | 52 (13.8) | 36 (15.7) | 78 (9.4) |  |
| Unknown | 9 (6.3) | 22 (5.8) | 5 (2.2) | 42 (5.1) |  |
| Glycaemic control, (%) |  |  |  |  | 0.072 |
| Normal | 123 (86.0) | 324 (85.9) | 207 (90.0) | 746 (89.8) |  |
| High | 14 (9.8) | 39 (10.3) | 20 (8.7) | 61 (7.3) |  |
| Unknown | 6 (4.2) | 14 (3.7) | 3 (1.3) | 24 (2.9) |  |
| NODAP, (%) | 1 (0.7) | 0 (0.0) | 0 (0.0) | 7 (0.8) | 0.393 |
| Glycaemic management, (%) |  |  |  |  | 0.147 |
| Without diabetes | 93 (65.0) | 269 (71.4) | 169 (73.5) | 585 (70.4) |  |
| ultra-fast insulins | 8 (5.6) | 24 (6.4) | 16 (7.0) | 65 (7.8) |  |
| Oral hypoglycemic drugs | 28 (19.6) | 55 (14.6) | 27 (11.7) | 114 (13.7) |  |
| Combinations of insulin and oral hypoglycemic drugs | 13 (9.1) | 26 (6.9) | 12 (5.2) | 53 (6.4) |  |
| Non-pharmacological intervention | 1 (0.7) | 3 (0.8) | 6 (2.6) | 14 (1.7) |  |
| Postoperative Diets, (%) |  |  |  |  | 0.880 |
| Sodium and fat-restricted diets | 135 (94.4) | 335 (88.9) | 213 (92.6) | 750 (90.3) |  |
| Higher Sodium and fat diets | 8(5.6) | 42(11.1) | 17(7.4) | 81(9.7) |  |
| Exercise, (%) |  |  |  |  | 0.029 |
| More than three times a week | 103 (72.0) | 290 (76.9) | 182 (79.1) | 679 (81.7) |  |
| Once in a while | 36 (25.2) | 85 (22.5) | 40 (17.4) | 147 (17.7) |  |
| Never | 4 (2.8) | 2 (0.5) | 8 (3.5) | 5 (0.6) |  |
| Medications, (%) |  |  |  |  |  |
| Aspirin | 123 (86.0) | 327 (86.7) | 197 (85.7) | 741 (89.2) | 0.325 |
| P2Y_12_ inhibitor | 123 (86.0) | 329 (87.3) | 206 (89.6) | 768 (92.4) | 0.003 |
| Statins | 118 (82.5) | 281 (74.5) | 176 (76.5) | 649 (78.1) | 0.693 |
| RAAS Inhibitors | 72 (50.3) | 172 (45.6) | 103 (44.8) | 362 (43.6) | 0.267 |
| β-blockers | 69 (48.3) | 178 (47.2) | 93 (40.4) | 368 (44.3) | 0.142 |

NODAP:New-Onset Diabetes after PCI; RAAS:Renin-angiotensin-aldosterone system; β-blockers:Beta-adrenergic receptor blockers.

**Table S4.**  **Results of Linear hypothesis test with the RCS function**

| Variable | χ^2^-value | df | *P*-value |
| --- | --- | --- | --- |
| Age |  |  |  |
| Overall-association | 39.65 | 3 | <.001 |
| Nonlinear-association | 1.87 | 2 | 0.392 |
| Number of stents |  |  |  |
| Overall-association | 6.87 | 2 | 0.032 |
| Nonlinear-association | 0.04 | 1 | 0.841 |
| LDL-C |  |  |  |
| Overall-association | 17.98 | 3 | <.001 |
| Nonlinear-association | 3.19 | 2 | 0.203 |
| LVEF |  |  |  |
| Overall-association | 27.30 | 3 | <.001 |
| Nonlinear-association | 12.80 | 2 | 0.002 |
| BUN |  |  |  |
| Overall-association | 2.20 | 3 | 0.532 |
| Nonlinear-association | 0.03 | 2 | 0.984 |
| eGFR |  |  |  |
| Overall-association | 4.65 | 3 | 0.200 |
| Nonlinear-association | 2.89 | 2 | 0.236 |
| TC |  |  |  |
| Overall-association | 4.48 | 3 | 0.214 |
| Nonlinear-association | 3.52 | 2 | 0.172 |
| ANC |  |  |  |
| Overall-association | 6.40 | 3 | 0.094 |
| Nonlinear-association | 0.25 | 2 | 0.884 |
| AST |  |  |  |
| Overall-association | 7.09 | 3 | 0.069 |
| Nonlinear-association | 4.12 | 2 | 0.128 |
| LAD |  |  |  |
| Overall-association | 2.13 | 3 | 0.546 |
| Nonlinear-association | 0.72 | 2 | 0.697 |
| MDP |  |  |  |
| Overall-association | 3.27 | 3 | 0.352 |
| Nonlinear-association | 0.53 | 2 | 0.766 |

Association between continuous variables and MACEs in the training set population (adjusted for Age, Killip Ⅲ-IV, Hypertension, HHcy, HF, Number of stents, multivessel disease, LDL-C, LVEF; The knot of the variable named "Number of stents" in the RCS was 3 and was set at the 10th, 50th, and 90th percentiles of the distribution, respectively.)


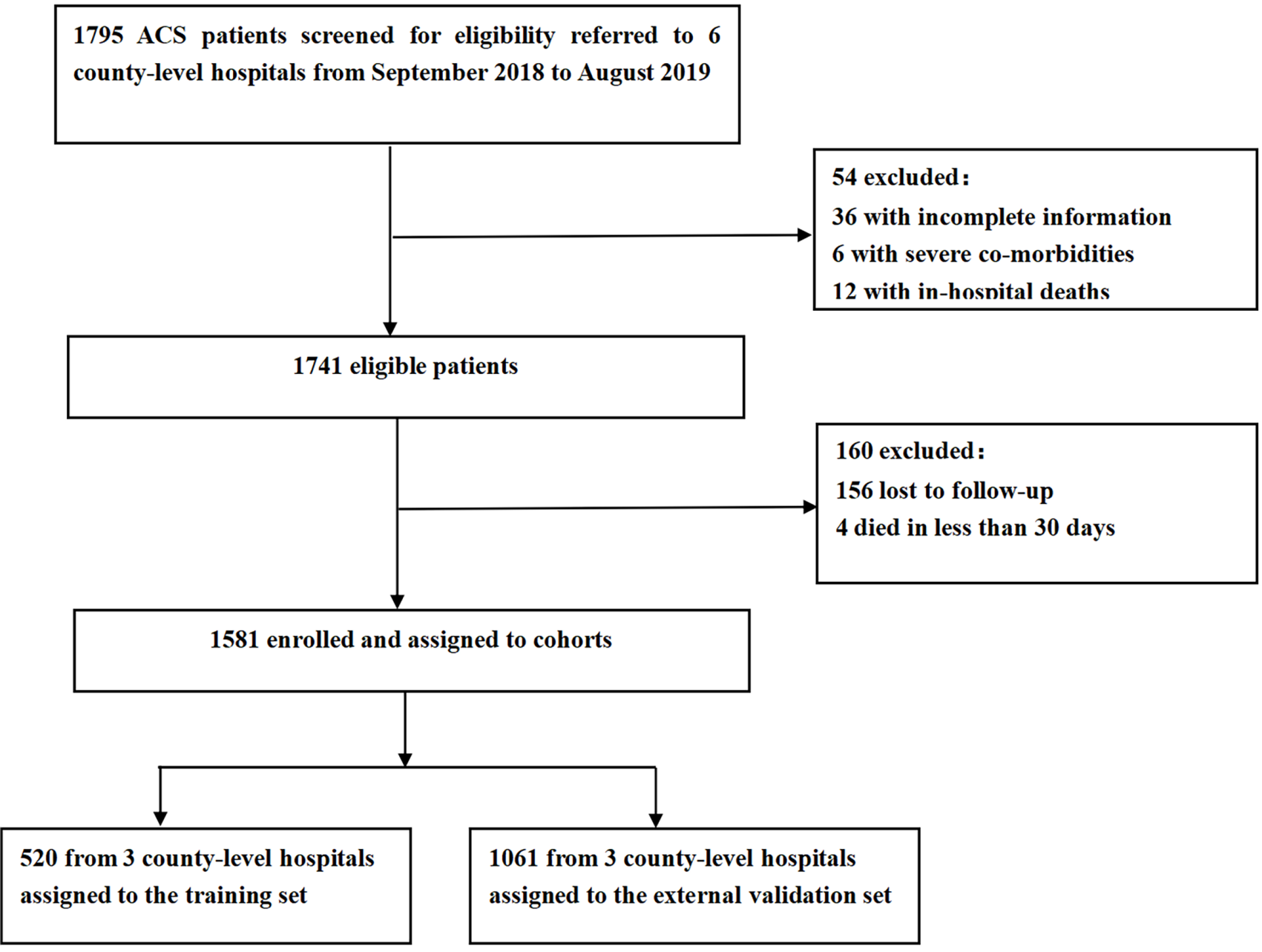


**Figure S2** Study flow diagram.


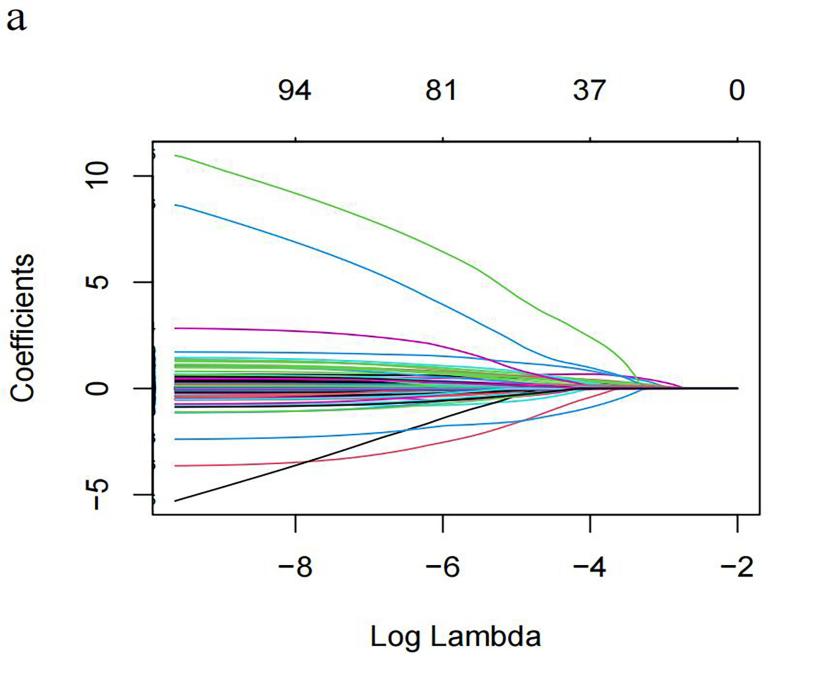

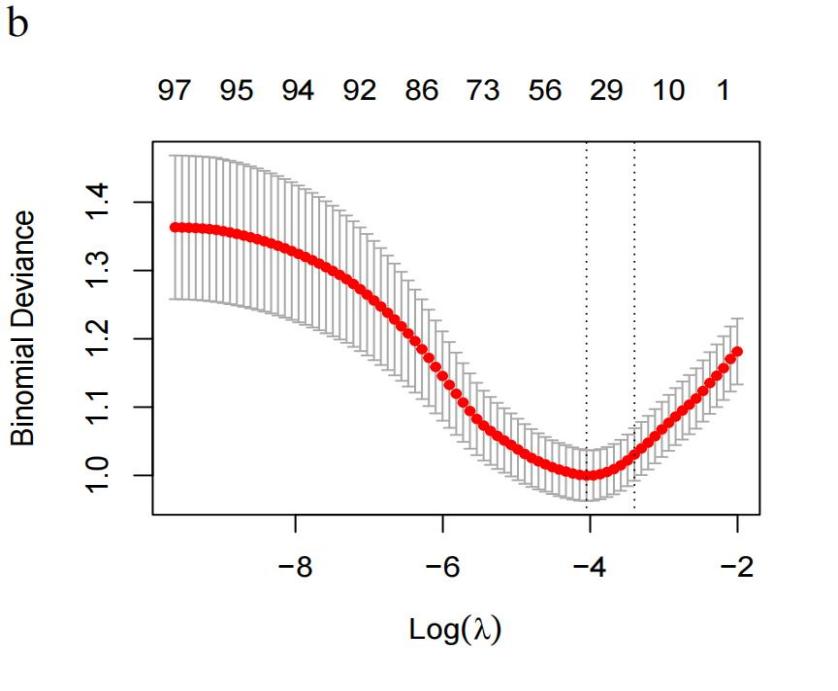


**Figure S3** Feature selection using the LASSO binary logistic regression model

**Notes**: (**a**) The figure shows the LASSO coefficient curves for 97 variables based on log(lambda). The distribution of the coefficients was generated by the sequence. (**b**) A vertical line was drawn at the value selected using ten-fold cross-validation. The optimal model was acquired when the lambda was 0.03340004, where the optimal lambda resulted in fifteen features with nonzero coefficients.


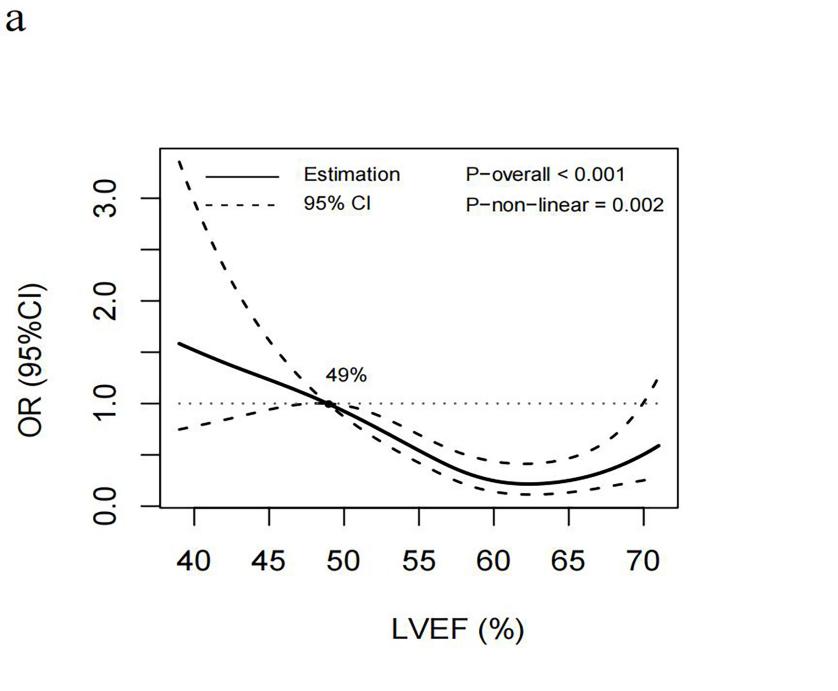

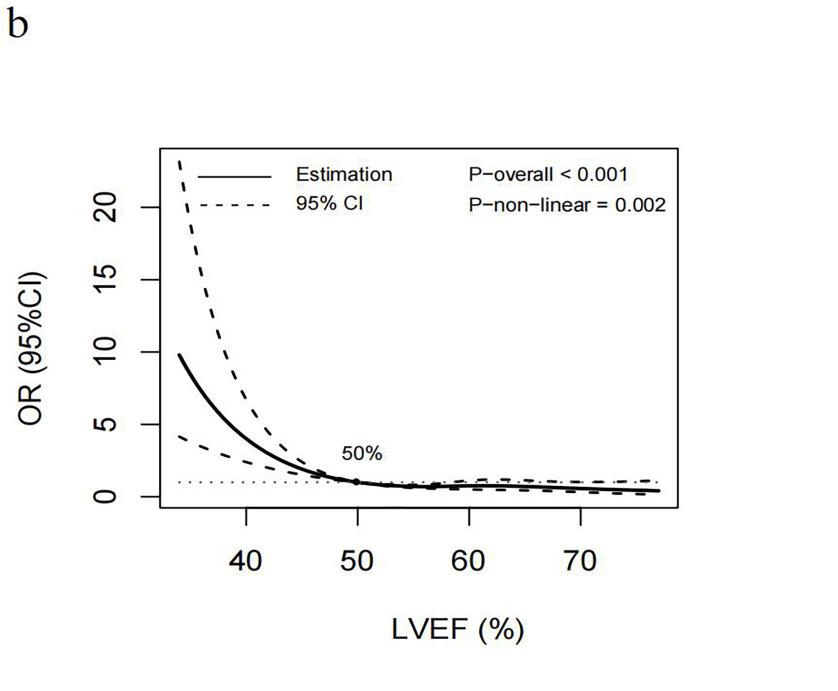


**Figure 4** Association of LVEF with risk of MACEs (**a** shows an association of LVEF with risk of MACEs after adjustment for other confounders in the training set; **b** shows an association of LVEF with risk of MACEs after adjustment for other confounders in the external validation set.)


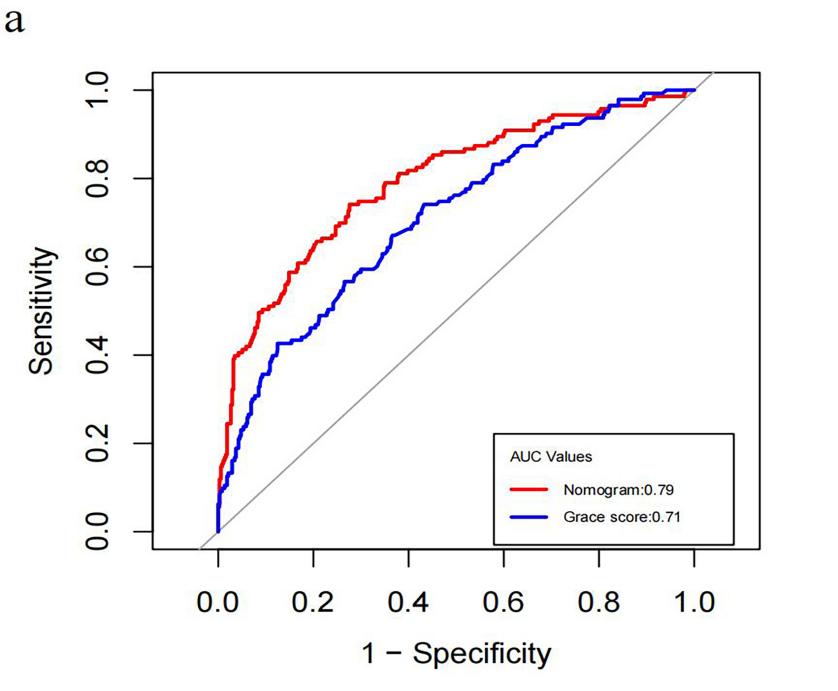

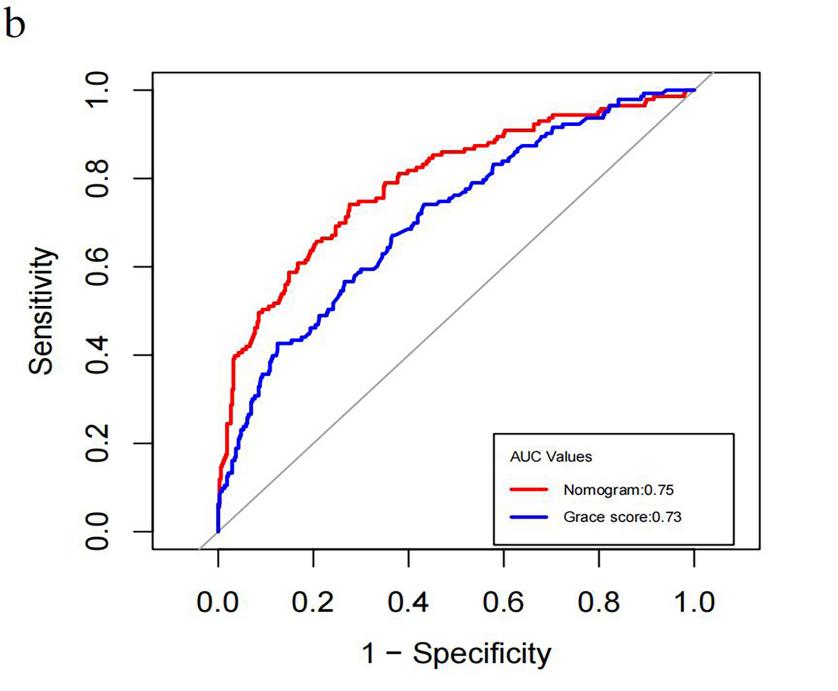


**Figure S5** ROC curve of the nomogram for predicting the risk of MACEs after PCI in ACS patients. (a) ROC curve

in the training set; (b) ROC curve in the validation set.


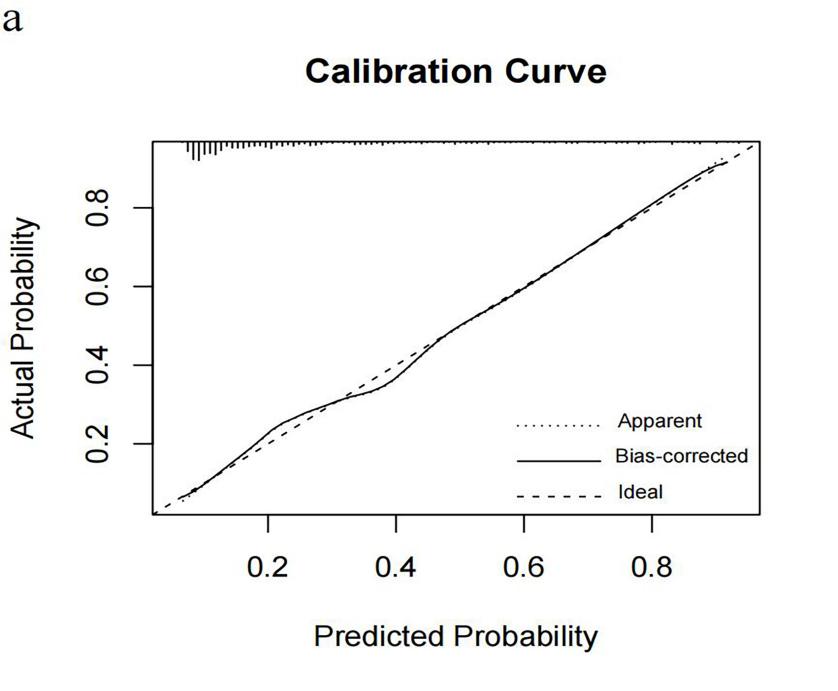

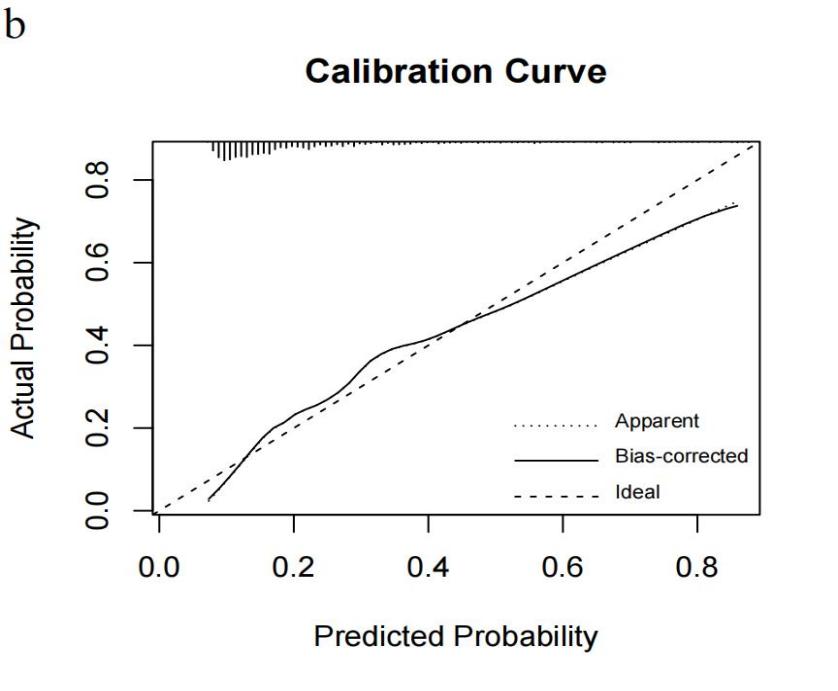


**Figure S6** Calibration curve of the nomogram for the training set (a) and the validation set (b). The X-axis represents

the overall predicted probability of MACEs after PCI, and the Y-axis represents the actual probability. Model calibration is indicated by the degree of fitting of the curve and the diagonal.


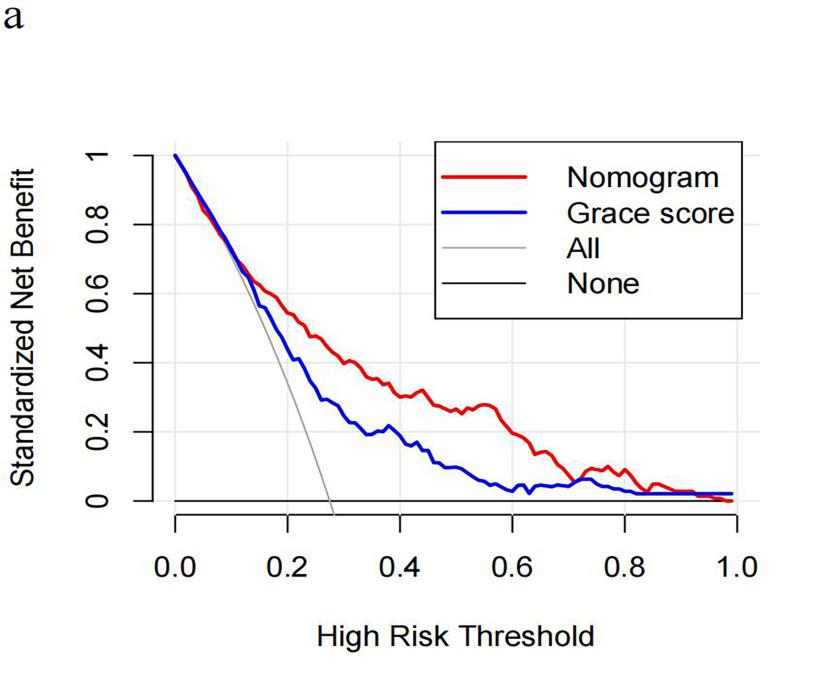

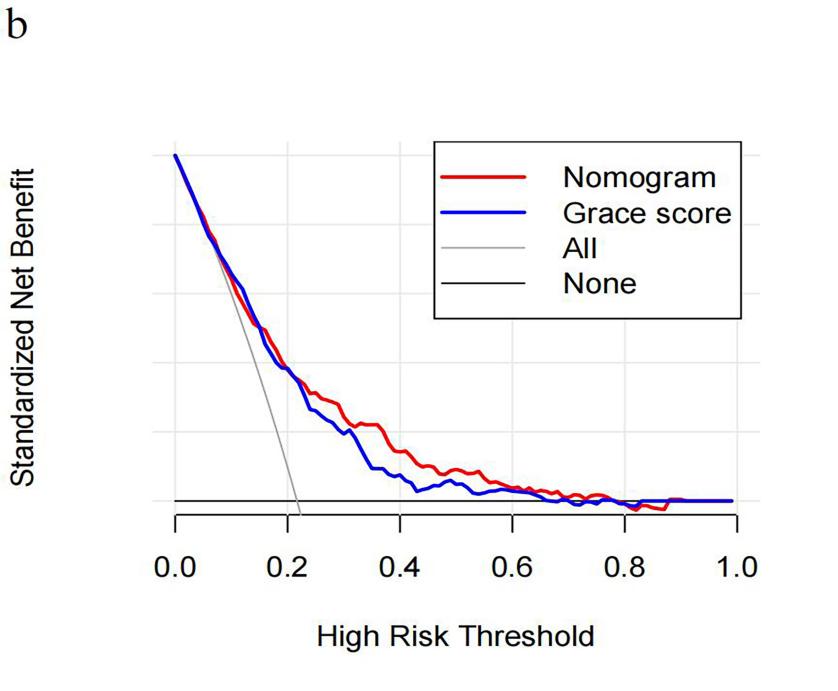


**Figure S7** Decision curve analysis (DCA) of the nomogram (a DCA of training set, b DCA of validation set)


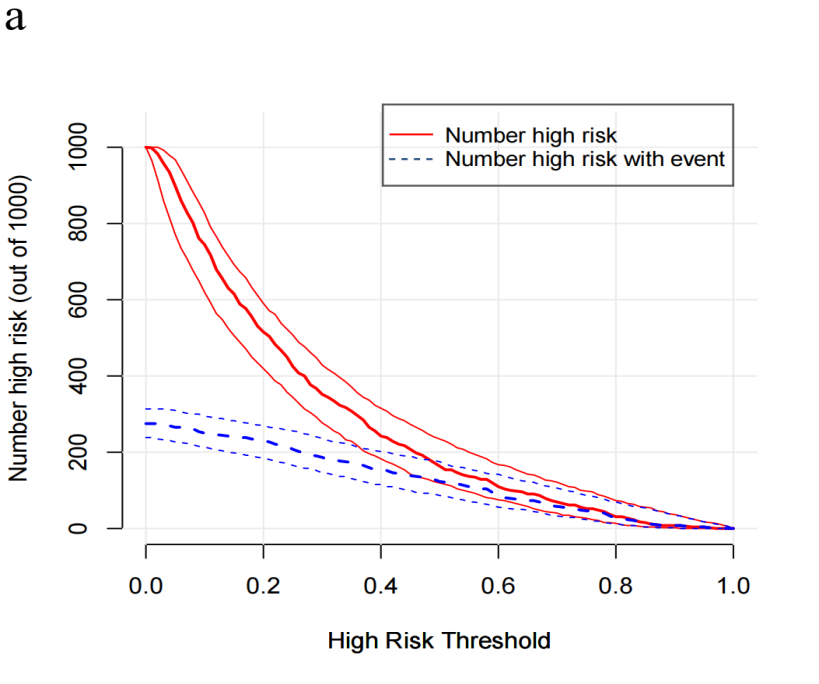

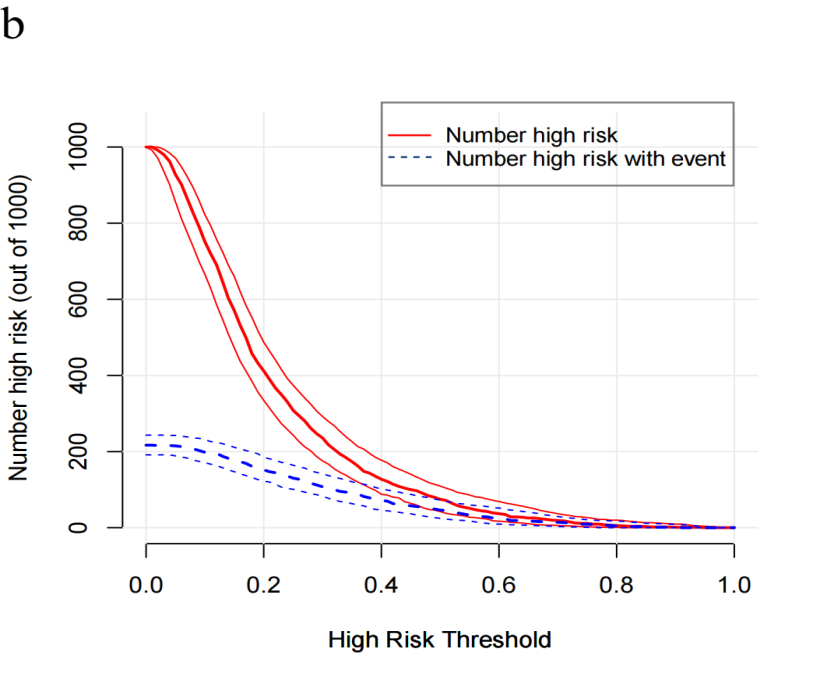


**Figure S8** Clinical Impact Curve (CIC) of the nomogram (a CIC of training set, b CIC of validation set)

**SUPPLEMENTARY REFERENCES**

1. [2019 Chinese Society of Cardiology (Csc) Guidelines for the Diagnosis and Management of Patients with St-Segment Elevation Myocardial Infarction]. *Zhonghua Xin Xue Guan Bing Za Zhi* (2019) 47(10):766-83. Epub 2019/10/28. doi: 10.3760/cma.j.issn.0253-3758.2019.10.003.

2. [Guideline and Consensus for the Management of Patients with Non-St-Elevation Acute Coronary Syndrome(2016)]. *Zhonghua Xin Xue Guan Bing Za Zhi* (2017) 45(5):359-76. Epub 2017/05/18. doi: 10.3760/cma.j.issn.0253-3758.2017.05.003.

3. Guidelines for the Diagnosis and Treatment of Unstable Angina Pectoris and Non-St-Segment Elevation Myocardial Infarction. *Zhonghua Xin Xue Guan Bing Za Zhi* (2007) 10(04):295-304. doi: 10.3760/j.issn:0253-3758.2007.04.003.

4. Tibshirani R. Regression Shrinkage and Selection Via the Lasso. *Journal of the Royal Statistical Society: Series B (Methodological)* (2018) 58(1):267-88. doi: 10.1111/j.2517-6161.1996.tb02080.x.

5. Efron B, Hastie TJ, Johnstone I, Tibshirani R. Least Angle Regression. *Annals of Statistics* (2004) 32:407-99.

6. Tibshirani R, Saunders M, Rosset S, Zhu J, Knight K. Sparsity and Smoothness Via the Fused Lasso. *Journal of the Royal Statistical Society Series B: Statistical Methodology* (2004) 67(1):91-108. doi: 10.1111/j.1467-9868.2005.00490.x.
